# Drug-induced Extrapyramidal Symptoms Scale (DIEPSS) Serbian Language version: Inter-rater and Test-retest Reliability

**DOI:** 10.1038/s41598-017-08706-3

**Published:** 2017-08-14

**Authors:** Ami Peljto, Ljubica Zamurovic, Milica Pejovic Milovancevic, Branko Aleksic, Dusica Lecic Tosevski, Toshiya Inada

**Affiliations:** 1 Institute of Mental Health, Palmoticeva 37, 11000 Belgrade, Serbia; 2 School of Medicine, University of Belgrade, Dr Subotica 8, 11000 Belgrade, Serbia; 3 Department of Psychiatry, Nagoya University Graduate School of Medicine, 65 Tsurumai-cho, Showa-ku, Nagoya, Aichi-ken 466-8550 Japan; 4 Office on International Affairs, Nagoya University Graduate School of Medicine, 65 Tsurumai-15 cho, Showa-ku, Nagoya, Aichi-ken 466-8550 Japan; 5 Serbian Academy of Sciences and Arts, Belgrade, Serbia; 6 Department of Psychiatry and Psychobiology, Nagoya University Graduate School of Medicine, 65 Tsurumai-cho, Showa-ku, Nagoya, Aichi-ken 466-8550 Japan

## Abstract

Drug-induced Extrapyramidal Symptoms Scale (DIEPSS) is developed in the era of second-generation antipsychotics and is suitable for evaluation of the low incidence of extrapyramidal symptoms occurring in the treatment of atypical antipsychotics, as well as the relationship between personal and social functioning. The study was carried out at the Institute of Mental Health in Serbia in 2015 Study used the 127 DIEPSS video clips material, recorded from 1987 till 2015. Four raters performed the assessment simultaneously, individually rating one assigned item immediately after seeing the video clip. For the purpose of evaluating test-retest reliability the second assessment of the same material was performed nine months after the first assessment. Inter-rater reliability was high for each individual item, with ICCs ranging from 0.769 to 0.949. The inter-rater reliability was highest for akathisia item and lowest for dyskinesia. The test-retest reliability was high for each individual item, with ICC ranging from 0.713 to 0.935. The test-retest reliability was highest for bradykinesia item and lowest for dystonia. The Serbian version of DIEPSS has high level of inter-rater and test-retest reliability. High values of concordance rates (ICC > 0.7) for each evaluated individual item suggest that items of DIEPSS are well defined.

## Introduction

Antipsychotics are used worldwide for the management of a number of mental illnesses. They are used to treat serious mental health conditions such as schizophrenia, bipolar disorders as well as other emotional and mental health conditions. In recent years, evidence from clinical trials has shown that antipsychotics are associated with significant adverse effects and that there is an urgent need for careful evaluation of their use^1–3^.

The use of antipsychotics may cause various spectrum of movement disorders, as well as metabolic and cardiac side effects that can lead to early treatment discontinuation as well as poor quality of life^2^. If these side effects are poorly controlled, they could dramatically increase the morbidity and the mortality^3^. Treatment monitoring, including effectiveness on the targeted symptoms, tolerance and observance, are major issues in the management of patients treated with antipsychotics.

Movement disorders are classified as separate diagnostic category in a section II of the classification system of mental disorders (Diagnostic and Statistical Manual of Mental Disorders 5th ed^4^) as Medication-induced movement disorders and other adverse effect of medication. However, in the ICD-10 movement disorders are recognized under the section of extrapyramidal symptoms as neurological disorders^5^. Drug-induced movement disorders (DIMD) have dramatically declined with use of SGA but remain important in clinical practice and for the understanding antipsychotic pharmacology^6^.

The presence of extrapyramidal symptoms has led to development of various rating scales for evaluation of iatrogenic-induced movement disorders^7^. Generally speaking, rating scales can be divided into those for use in clinical research and those for use in every day clinical practice. Additionally, these scales can be focused exclusively on particular symptoms such as parkinsonism (The Simpson-Angus Scale–SAS)^8^, akathisia (the Barnes Akathisia Rating Scale–BARS)^9^, or dyskinesia (the Abnormal Involuntary Movement Scale–AIMS)^10^. On the other side, there are combined scales for evaluation of global extrapyramidal symptoms (the Extrapyramidal Symptom Rating Scale–ESRS)^11^ or combination of rating scales for individual symptoms (SAS, BARS and AIMS).

Drug-induced Extrapyramidal Symptoms Scale (DIEPSS) is a multidimensional scale combining rating scale designed for assessing drug-induced extrapyramidal symptoms^12^. The DIEPSS is developed in the era of SGA and is suitable for evaluation of the low incidence of extrapyramidal symptoms occurring in the treatment of atypical antipsychotics^13, 14^. Nowadays, it is widely used for evaluation of drug-induced extrapyramidal symptoms, as well as the relationship between personal and social functioning^15^. It consists of eight individual items and one global domain^12, 14, 16^, each rated on 5-point scale^12^. While some multi-domain scales could be difficult to score, the DIEPSS scale belongs to group with the most valid, reliable, and easy-to-use scales for use in clinical practice^17^. Moreover, this scale has good psychometric characteristics^18^.

The use of first generation antipsychotics (FGA) has declined in the last few years, not because of the increase in prescriptions of second-generation agents (SGA), but because of the side effects that FGA cause very frequently. Despite of the discovery of the atypical antipsychotics and their favorable extrapyramidal symptom profile, many studies have shown that some patients are at still at high risk of extrapyramidal symptomatology^19^. Therefore, clinicians should be aware of the emergency of extrapyramidal symptoms all the time. With all these factors in mind the DIEPPS scale was developed for screening extrapyramidal symptoms as practical and standardized rating scale in a simple and easy manner for administration.

The aim of this study was to validate the level of inter-rater and test-retest reliability of DIEPPS scale.

## Methods

### Material

The study was carried out at the Institute of Mental Health in Serbia in 2015. Serbian version of the DIEPSS was created through translation and back-translation from the original English version, with permission of the author. Study used the DIEPSS video recorded training material, which was first established in 2001^14^. Material was used with kind permission of the author. It consisted of 127 video clips, recorded from 1987 till 2015. The subjects recorded were Japanese psychiatric patients receiving first and/or second generation antipsychotics. Total number of patients who appeared in the video clips was 106, 47 male and 59 female. Mean age was 48.1 ± 16.3 years (range 18–80) at the time of video recording. Subjects were diagnosed with schizophrenia (69 patients) and mood disorders (37 patients). The ethics committee of Seiwa hospital, Institute of Neuropsychiatry, Tokyo, Japan, approved the experimental protocols used in the current study. All experiments were performed in accordance with the committees’ guidelines and regulations. Written informed consent was obtained from all participants or legal guardians. Each patient’s capacity to provide consent was confirmed by a family member or legal guardians when needed.

### Assessments

The DIEPSS is composed of 9 items, 8 individual and one global item. Individual items are gait, bradykinesia, sialorrhea, rigidity, tremor, akathisia, dystonia and dyskinesia. Evaluation of individual items is principally based on objective observations. Global item, overall severity, considers severity and frequency of individual items, subjective distress and influence on daily activities. Each item is rated from normal (0) to severe (4)^12, 14^.

In this study we used individual items of DIEPSS. Global item was excluded considering study design that implied using video clips.

### Evaluation of Serbian version of DIEPSS

Four trained psychiatrists, two male and two female, participated in the study. Before the initiation of the study the session was held for all raters to clarify the procedure and to distribute scale and instructions. Raters performed the assessment simultaneously, individually rating one assigned item immediately after seeing the video clip. Raters were blind to one another during assessments. For the purpose of evaluating test-retest reliability the second assessment of the same material was performed nine months after the first assessment. Evaluation of inter-rater reliability was held on September 22 & 23, 2015 in Belgrade, Serbia; evaluation of test-retest reliability was held on August 10 &17, 2016 in Nagoya, Japan.

### Data analysis and statistics

The inter-rater and test-retest reliability were analyzed using Analysis of Variance Interclass Correlation Coefficient (ANOVA ICC)^20^ for each evaluated item and for each pair of raters. ANOVA ICCs were calculated using two methods independently: EXCEL, with calculation formula input according to manuscript by Bartko and Carpenter^21^, and SPSS version 24 program.

## Results

Results of the inter-rater reliability are shown in Table 1. Inter-rater reliability was high for each individual item, with ICCs ranging from 0.769 to 0.949. The inter-rater reliability was highest for akathisia item and lowest for dyskinesia.

**Table 1:** Inter-rater reliability of DIEPSS Serbian version.

DIEPSS item	ANOVA ICC		Number of		Rating range	
	SPSS	(95% Confidence Interval)	Raters	Patients	MIN	MAX
1. Gait	0.856	(0.744–0.933)	4	20	0	4
2.Bradykinesia	0.891	(0.801–0.949)	4	20	0	4
3. Sialorrhea	0.842	(0.724–0.924)	4	21	0	4
4. Muscle rigidity	0.948	(0.904–0.976)	4	22	0	4
5. Tremor	0.775	(0.636–0.882)	4	24	0	4
6. Akathisia	0.949	(0.907–0.976)	4	22	0	4
7. Dystonia	0.925	(0.868–0.963)	4	24	0	4
8. Dyskinesia	0.769	(0.623–0.880)	4	23	0	4

Results of the test-retest reliability are shown in Table 2. The test-retest reliability was high for each individual item, with ICC ranging from 0.713 to 0.935. The test-retest reliability was highest for bradykinesia item and lowest for dystonia.

**Table 2:** Test-retest reliability of DIEPSS Serbian version.

DIEPSS item	ANOVA ICC		Number of		Rating range	
	SPSS	(95% Confidence Interval)	Raters	Patients	MIN	MAX
1. Gait	0.924	(0.822–0.969)	2	20	0	4
2.Bradykinesia	0.935	(0.846–0.974)	2	20	0	4
3. Sialorrhea	0.746	(0.479–0.888)	2	21	0	4
4. Muscle rigidity	0.862	(0.702–0.940)	2	22	0	4
5. Tremor	0.717	(0.453–0.866)	2	24	0	4
6. Akathisia	0.772	(0.532–0.898)	2	22	0	4
7. Dystonia	0.713	(0.446–0.864)	2	24	0	4
8. Dyskinesia	0.723	(0.456–0.872)	2	23	0	4

## Discussion

Nonadherence to medication can have negative consequences for the patient, the provider, the physician, and even the researchers who are working to establish the value of the medication for the target population. It is a common problem induced by many factors, including the medication side effects. Nonadherence increases the risk of relapse, rehospitalization, and self-harm, increases inpatient costs, and lowers quality of life of patients. The efficacy of antipsychotic medication in the acute and maintenance treatment of several mental disorders is clear from large meta-analyses of placebo-controlled trials^22^.

Data from the CATIE trial imply that advantages of SGA in significantly reducing extrapyramidal side effects compared with FGA may be diminished when compared with modest doses of lower-potency first generation drugs. However, the dichotomy between FGA and SGA may be oversimplified, and CATIE trial showed that antipsychotics could be conceptualized as a single drug class with a spectrum of risk for movement disorders depending upon receptor binding affinities and individual patient susceptibility^7^. Especially, the use of SGAs for populations that were drug naive (children, adolescents, elderly) and adult population with conditions such as bipolar disorder and major depressive disorder, together with a lack of long-term clinical trials that would precisely indicate the rate of DIMD in these populations, portends an increase in the actual number of cases^23^.

All mentioned are reasons to develop instruments for easy and fast assessment of side effects, especially drug-induced movement disorders, experienced by patients as very unpleasant and potentially with high nonadherence potential.

Ethnical differences were suggested as possible factor that could alter susceptibility to adverse drug reactions, including extrapyramidal side effects. Ethnic groupings may account for some of the complex interactions between genetics, environment and society^24^. There are results that suggest that East Asian patients have a significantly greater risk of drug induced extrapyramidal symptoms compared with non-East Asian patients^24^. Many studies of assessment of drug induced extrapyramidal syndrome with DIEPSS were conducted in East Asian population^13, 14, 25^. Assessment of drug induced extrapyramidal syndrome with DIEPSS in Serbian and other non-East Asian populations could contribute to better understanding of potential differences in incidence and severity of drug induced extrapyramidal syndrome among different populations.

Our results show that Serbian version of DIEPSS has high level of inter-rater and test-retest reliability. High values of concordance rates (ICC > 0.7) for every evaluated individual item suggest that items of DIEPSS are well defined. High reliability that is shown could also be related to the use of video recorded material, which provided consistence and stability for rated items. Very high level of inter-rater reliability for akathisia and muscle rigidity (ICC value 0.949 and 0.948 respectively) is a significant result, considering that assessment of subjective distress of patients was not possible and no physical examination could be performed. These findings confirm that subjects for DIEPSS training material are well chosen. Present findings are in concordance with previous results confirming that DIEPSS is a valid and reliable measure of extrapyramidal symptoms^2, 13^.

## Limitations

This study excluded global item since study design did not provide assessment of subjective distress. Hence, concordance rates for total score of DIEPSS couldn’t be calculated. For future research it would be important to evaluate concurrent validity of Serbian version of DIEPSS, comparing it with other rating scales for extrapyramidal symptoms.

## Conclusion

In conclusion, the results of our study show that the DIEPSS is reliable instrument for identifying extrapyramidal symptoms and it can be used in clinical practice to improve early detection, assessment and treatment.
